# The Effect of Assisted Reproductive Technologies on Gynecological Cancer: Report of Our Experiences and Literature Review

**Published:** 2013-09

**Authors:** Mojgan Karimi Zarchi, Mitra Rouhi, Alime H. Abdolahi, Seyedhossein Hekmatimoghaddam

**Affiliations:** 1 Department of Gynecology Oncology, Shahid Sadoughi University of Medical Science, Yazd, Iran;; 2 Faculty of Medical Sciences, Islamic Azad University, Yazd Branch, Yazd, Iran;; 3 Department of Laboratory Sciences, Shahid Sadoughi University of Medical Science, Yazd, Iran

**Keywords:** Assisted reproductive technologies, Infertility, Ovarian cancer, Endometrial cancers, Breast cancers, Age, Body mass index

## Abstract

**Introduction:**

Infertility is as an important and common problem in couples necessitating assisted reproductive technology (ART) or drug therapy. Infertility is known as a risk factor for ovarian, breast and endometrial cancer. We aimed on evaluation of the history of primary infertility and previous ART in patients with the above-mentioned cancers.

**Material and method:**

In this retrospective study we evaluated all of the risk factors in patients with breast cancer, ovarian cancer and endometrial cancers who referred to the Gynecological Oncology Clinic in Shahid Sadoughi Hospital in Yazd, Iran from 2002 to 2012. We also investigated the history of primary infertility and ART in these patients before diagnosis of cancers.

**Results:**

We gathered data from 92 patients with endometrial cancer, 84 patients with advanced epithelial ovarian cancer and 113 patients with breast cancer. There was history of infertility in 39.1% of patients with endometrial cancer who were obese (body mass index, BMI>29) and in 18.8% of patients with endometrial cancer and normal body mass index (BMI=25-29). ART had been performed in 7.3% of all patients with endometrial cancer. In patients with epithelial ovarian cancer, infertility was diagnosed in 28.4% and ART applied in 14.1%. Clomiphene with or without HCG and HMG was the most common drug used for patients with ovarian cancer. In patients with breast cancer, there was infertility in 16.5% and ART performed in 7.3%.

**Conclusion:**

Although infertility was present as an important and fairly common risk factor in some patients with endometrial, ovarian and breast cancer, but some other factors may be more important, including age, BMI and the etiology of infertility. Finding the association between ART and gynecological cancers needs long cohort studies with follow-up of infertile women who get the ART or drug therapy for over 15-20 years. We think BMI and age (in addition to infertility and ART) are contributing factors for development of gynecological cancers.

## INTRODUCTION

Nowadays women have more opportunity for a successful pregnancy with existence of assisted reproductive technologies (ART) which augment circulating estrogens. However, the danger of hormone-sensitive malignancies like breast, ovarian and uterine cancers increase by using such treatments (1). The aim of this study was investigation of the effect of infertility and ART on gynecological cancer and also report of our experiences with patients who referred to a gynecological oncology center in Yazd, Iran.

## MATERIALS AND METHODS

We evaluated all of the known risk factors in patients with breast cancer, ovarian cancer and endometrial cancer who referred to the Gynecological Oncology Clinic in Shahid Sadoughi Hospital within 2002-2012. In a retrospective study, we investigated the history of primary infertility and any type of ART before diagnosis of cancers in those patients. Data were analyzed by the software SPSS 11. Also we made a comprehensive research on all of the papers which were indexed in Pubmed-Medline between 2000 and 2013 with the key words of infertility, ART, gynecological cancer, and breast cancer.

## RESULTS

We gathered data from 92 patients with endometrial cancer, 84 patients with advanced epithelial ovarian cancer and 113 patients with breast cancer. There was history of infertility in 39.1% of patients with endometrial cancer who were obese (body mass index, BMI>29) and in 18.8% of patients with endometrial cancer and normal body mass index (BMI=25-29). ART had been performed in 7.3% of all patients with endometrial cancer. In patients with epithelial ovarian cancer, infertility was diagnosed in 28.4% and ART applied in 14.1%. Clomiphen with or without HCG and HMG was the most common drug used for patients with ovarian cancer. In patients with breast cancer, there was infertility in 16.5% and ART performed in 7.3%.

## DISCUSSION

Generally, we found significant association of primary infertility (as a common known risk factor) with gynecological cancers, but in the case of ART there is need for a large cohort study and long follow-up of the patients. Our patients clearly showed the higher incidence of all 3 major types of gynecological malignancies as compared with their much lower incidence in the naturally fertile female population. The association between gynecological cancer and *in vitro* fertilization (IVF) or ART is discussed below.

### Breast cancer

Breast cancer is the most common cancer in women in the US, the second leading cause of cancer death in women, and the most common cause of death in women aged 40 to 59. The known risk factors, such as age at menarche, first live birth, menopause, and proliferative breast disease are present in about 50% of cases. Another 10 percent give a positive family history (1).

A relationship between menarche, menopause, and breast cancer risk has been found in epidemiologic studies. These events influence a woman’s cumulative exposure to ovarian hormones by changing the number of lifetime ovulatory cycles. In fact, the inverse association between obesity and breast cancer risk in premenopausal women may be explained by ovulatory abnormalities. Some researchers say that there may be a link between infertility due to anovulatory disorders and a decreased risk of breast cancer (2-4). Pregnancy factors (such as the age at first pregnancy, the number of fetuses in each pregnancy, and history of any abortion or gestational trophoblastic disease) affect breast cancer risk, and multiple births decrease its risk (5). Delivering a baby in older age will increase risk of developing breast cancer. A majority of such women seek infertility management methods and use hormonal drugs that increase the risk of breast cancer (6). Overall, the relationship between infertility treatment and risk of breast cancer is not clear (7-17). However, only a single study in 2005 by Burkman *et al*. showed that gonadotropins increase risk of this cancer (18). Multiple births are common after IVF, but when comparing women who had twin delivery following IVF and other women, it seems that breast cancer is lower than expected after IVF (19, 20). Katze *et al*. in 2008 showed that breast cancer has direct relationship with the age at IVF (21). Long time exposure to endogenous estrogen increases the risk of breast cancer. Estrogen subtypes such as estriol and estradiol control the ovarian function. After menopause, the main source of estrogen is dehydroepiandrosterone (DHEA). Different ways of suppressing ovarian function or using drugs for reduction of estrogen level decreases breast cancer risk (22). Serum estrogen increases with clomiphene citrate and FSH/hCG during infertility treatment (23). The roles of progestins, prolactin and insulin are less established but one study showed that risk of ductal breast cancer increases ∼4 fold after use of progesterone (10). Long period of estrogens exposure, including early menarche and late menopause, is a well-known risk factor for breast cancer (24, 25). The direct effect of infertility treatment agents on mammary epithelial and/or stromal tissue, independent of downstream estrogen elevation, is not known. Serum estrogen levels are greater during cycles in which ovulation is induced than in natural cycle in women. Moreover, ovulation of multiple follicles results in high progesterone levels, which may also increase breast cancer risk.

Clomiphene citrate acts by binding to estrogen receptor (ER), and exerts a weak estrogenic effect while blocking endogenous estrogen (26). Potential mechanism of action of clomiphene citrate may be through estrogenic transcriptional activity, and these influences are possibly dependent on its dose, endogenous tissue metabolism and the hormonal environment (27).

The association between clomiphene citrate exposure and breast cancer risk has been shown in several studies, but a recent meta-analysis did not support this conclusion, due to lack of long-term follow-up for most of the previous studies (28). While hCG exists in high levels in early pregnancy, both anti-invasive and anti-proliferative effects may be involved through the breast epithelial LH/hCG receptor and downregulation of nuclear factor-kappa B and activator protein-1 transcription factors in human breast cancer (29). It is important to mention that hCG has a major impact on reducing cell proliferation, irrespective of the type of cell or the hormone receptor status (6). The potential association between fertility drugs and breast cancer risk is not very clear. Gonadotrophins may raise estrogen levels during the follicular phase of ovulation induction cycles, but do not have any direct influence on breast tissue (10). In his research in 2010 Petru mentioned that premenopausal patients have a higher prevalence of BRCA gene mutation than older patients. So, in cases in whom the ovaries are preserved, the danger of cancer development in the ovary may be high. The premature ovarian failure is not blocked by normal menstrual cycle after chemotherapy/antihormonal therapy. It is suggested that, after antineoplastic therapy, the physician should wait for at least 2 months before evaluation of ovarian function. For testing the ovarian reserve, the most trustful factor is the anti-Muellerian hormone (AMH; Muellerian inhibiting factor, MIF). The protective influence of the GnRH analogue goserelin on ovarian procedure can be seen in four randomized studies which have explored it.

Observing various results, treatment of GnRH analogues together with cytostatic chemotherapy has to be regarded experimental at present, and cannot be suggested. In emergency cases at the time between breast cancer diagnosis and the start of chemotherapy, fertilization procedure may be performed. In such cases extracorporeal fertilization may be accomplished and embryo cryopreserved for preserving fertility (30). Using a gonadotrophin-releasing hormone agonist (GnRHa) trigger instead of hCG would decrease exposure to estrogen and make better cycle results. It results in better outcome by augmenting the yield of mature oocytes and embryos in aromatase inhibitor cycles, and furthermore it reduces the post-trigger estradiol exposure and ovarian hyperstimulation syndrome (OHSS) risks in women with breast cancer, too (31). Women who have been treated with IVF, have an increasing risk of having breast or uterine cancer in their early period after treatment especially the first year. An elevated risk of ovarian or uterine cancer was seen parallel to infertility, without any explained reason (32). The available information published in articles do not propose higher rate of breast cancer in women under fertility treatment. Unfortunately, some defects in these published data (like lack of enough follow-up) have put these studies under question seriously (33). At the present time, no proved evidence exists that supports relation between infertility drugs and any type of gynecological cancer. Mainly, women who are not fertile have more opportunity for gynecological malignancies. Nulligravidas that get treatment have higher risk of malignancy in comparison with women who had conceived after treatment. Studies (with limited evidence) have shown that clomiphene citrate could increase danger of ovarian and breast cancer when used 900 mg or over 6 cycles for treatment of women older than 40 years. But for an accurate and stable claim, results of more research with proper analytical and statistical procedures and follow-up time are needed to measure and evaluate the long-term impact of drugs discussed above (34).

### Ovarian cancer

Ovarian cancer is the second (after cervical carcinoma) most frequent gynecological cancer complicating pregnancy. Risk factors of ovarian cancer include familial history, past oral contraceptive use, past pregnancy, infertility, nulligravity, past breast feeding, and tubal ligation. Pregnancy factors affect ovarian cancer. Pregnancy decreases the risk for ovarian cancer but high age of mother at first pregnancy increases risk of ovarian cancer (35-37). Van Leeuwen *et al* showed a 2-fold increased risk of ovarian malignancies in women treated with ovarian stimulation for IVF compared with subfertile women not treated with IVF (19). A case–cohort study (38) showed an 11-fold increased risk of ovarian malignancies after 12 or more cycles of clomiphene. However, two cohort studies in Australia and Israel that did not show increased risk of ovarian cancer in the IVF group compared with the general population (14) while the recent Swedish study reported increased risk of ovarian cancer after IVF for parous women compared with all other Swedish women who gave birth in the study period (19). Crosbie *et al* said use of fertility drugs has little effect on ovarian cancer risk (39) but some studies indicated an increased risk in ovarian cancer (40, 41). This higher risk is lower than that seen before IVF (19). Development of ovarian tumors during or after ovarian stimulation treatment in some cases proposes this conclusion that ovarian stimulation may cause growth in already existed tumors (17, 42-44). Ayhan *et al* performed a study in 2004 that indicated no increase in risk of breast, uterine and invasive ovarian cancers, but the risk of borderline ovarian tumors might increase after such therapy (45). Studies from Australia (16) indicated a transient increase in risk of breast or uterine cancer in the first year after treatment, but the overall incidence of breast, ovarian or uterine cancers did not increase. In studies from Israel (12) no evidence of an increased cancer risk after IVF was found (14). However, after exclusion of cases which appeared within 1 year after IVF, the risk estimate declined and lost statistical significance. In a previous study from Sweden on women who underwent IVF (46) the researchers did not found increased risk for cancer, especially ovarian cancer, which was already increased prior to the first IVF treatment. Kurta *et al* in 2012 said that their results provide further evidence that fertility drug use does not significantly contribute to ovarian cancer risk among the majority of women; however, women who remain nulligravid despite infertility evaluation and fertility drug use, may have an elevated risk for it (47). Nowadays, the overall incidence of ovarian cancer is very low (one in 12500-25000 pregnancies), but the routine use of ultrasound in pregnancy has led to more frequent finding of adnexal masses (48).

### Endometrial cancer

Endometrial carcinoma is the most common female pelvic malignancy in developing countries, accounting about 7300 death in USA each year. It usually occurs after menopause but it has been reported that 3-5% of patients are younger than 40 years old. A history of ovarian dysfunctions, anovulation, obesity, nulliparity, hormonal disturbances or infertility can be found in most of these patients. They also show strong desire to keep their fertility (49).

Differences in epidemiology and prognosis suggest that two forms of endometrial cancer exist. Type I endometrial carcinoma is estrogen-related, usually presents histologically as a low-grade endometrioid tumor, and is associated with atypical endometrial hyperplasia. Type II endometrial carcinomas appear unrelated to estrogen stimulation or endometrial hyperplasia, and tend to present with higher grade tumors or poor prognostic cell types, such as papillary serous or clear cell tumors. Risk factors of endometrial cancer are tamoxifen use, diabetes, hypertension, chronic anovulation, exogenous estrogen, endogenous estrogen, obesity, age, Lynch syndrome, familial predisposition and genetics, breast cancer, early menarche and late menopause. Early-stage endometrial cancer is treated with hysterectomy and bilateral salpingo-oophorectomy. An emerging issue among younger affected women is the possibility of a fertility-sparing treatment (50). Using ART in these patients not only increases the opportunity of pregnancy but also makes pregnancy waiting time shorter.

Pregnancy is a main natural treatment, since the endocrine placenta is a source of natural continuous progestins. Also we have rare report cases of endometrial cancer during pregnancy, which shows that they hardly co-exist. Using ART ovulation induction drugs increases release of estrogens in these patients’ body, and is considered a risky concern in this field. However, there isn’t any report of higher risk of endometrial cancer after ovulation induction in literature. As we can see in Ricciardi *et al* study, there was no risk for a woman treated with clomiphene citrate before administering a progestin. Their data mention that women who were treated with progestin during 8 weeks of diagnosis of typical hyperplasia, were less exposed to the danger of developing endometrial carcinoma in comparison with untreated ones (50). Ovulation induction drugs used after conservative treatment of endometrial cancer did not increase the recurrence of endometrial cancer. Therefore, the resulting pregnancy seems to have a beneficial effect on the oncologic outcome (51). In patients undergoing infertility treatment an increased risk of endometrial cancer has been reported, which is expected based on similar structure shared by clomiphene and tamoxifen (52).

## CONCLUSION

The main contribution of this report is better knowledge about the higher incidence of 3 main types of gynecological cancers among infertile or ART-receiving women. Although infertility was diagnosed as an important and fairly common risk factor in endometrial, ovarian and breast cancers, but some other factors may be more important. Age, body mass index and the etiologic mechanism of infertility are also implicated. Finding the association of ART to gynecological cancers needs some long cohort studies with follow up of the infertile women who get the ART or drug therapy for over 15-20 years. Other types of studies in this field cannot answer our question about increased risk of gynecological cancer due to ART. We think BMI and age (in addition to infertility and ART) are contributing factors for development of gynecological cancers.
